# Exercise in preventing falls for men with prostate cancer: a modelled cost-utility analysis

**DOI:** 10.1007/s00520-022-06900-2

**Published:** 2022-02-24

**Authors:** Kim Edmunds, Paul Scuffham, Robert U. Newton, Daniel A. Galvão, Haitham Tuffaha

**Affiliations:** 1 Centre for the Business and Economics of Health, University of Queensland, Brisbane, QLD 4072 Australia; 2 Menzies Health Institute Queensland, Griffith University, Gold Coast, Australia; 3 Exercise Medicine Research Institute, Edith Cowan University, Joondalup, Australia

**Keywords:** Economic evaluation, Cost-utility analysis, Exercise medicine, Physical activity, Prostate cancer, Androgen deprivation therapy

## Abstract

**Introduction:**

Men who receive androgen deprivation therapy (ADT) for prostate cancer (PCa) are a vulnerable falls population due to the side effects of treatment. The purpose of this paper is to determine the cost-effectiveness of exercise in preventing falls and fractures for this high-risk population in Australia.

**Methods:**

A decision analytic model was constructed to evaluate the cost utility of an exercise intervention compared to usual care from a health system perspective. The intervention comprised two 1-h sessions of supervised exercise per week over 1 year for men with non-metastatic PCa receiving curative radiation therapy and ADT. A Markov model simulated the transition between five health states: (1) at risk of falling; (2) at recurrent risk of falling; (3) fracture (minor or major); (4) non-fracture injury (minor or major); and (5) death. Model inputs including transition probabilities and utility scores were obtained from published meta-analyses, and costs were drawn from Australian data sources (e.g. Medical Benefits Schedule). The model time horizon was 3 years, and costs and effects were discounted at 5% annual rate. Costs and quality-adjusted life years (QALYs) were aggregated and compared between the intervention and control to calculate incremental net monetary benefit (iNMB). Uncertainty in the results was explored using deterministic and probabilistic sensitivity analyses (PSA).

**Results:**

At a willingness-to-pay of AU$50,000 per QALY, the exercise intervention dominated, as it was less costly and more effective than usual care. The iNMB was $3010 per patient. The PSA showed a 58% probability the intervention was cost-effective.

**Conclusion:**

This is the first modelled economic evaluation of exercise for men with PCa. Our results suggest supervised exercise is cost-effective in reducing the risks of falls and fractures in this population.

**Supplementary Information:**

The online version contains supplementary material available at 10.1007/s00520-022-06900-2.

## Introduction

In Australia, over 80% of men with prostate cancer (PCa) are diagnosed with Stage I (localised) or II (locally advanced) disease [1] and have a 5-year survival rate of almost 100% [2]. For these men, this can mean dealing with the adverse effects of treatments such as androgen deprivation therapy (ADT) for many years. ADT medically suppresses the production of androgen and is associated with a number of adverse effects that are components of frailty such as muscle loss, reduced muscle strength, walking speed or cardiorespiratory fitness [3]. These adverse effects, through impaired physical function and associated fatigue [4], place patients and survivors of PCa at high risk of falls [5]. Another adverse effect of ADT is bone loss, which contributes to a high risk of fractures in this population. Studies of men receiving ADT report significant bone mineral density (BMD) declines at all sites in the first year (ranging from 1.8 to 6.5% at the femoral neck and 2 to 8% at the lumbar spine) [6], which progress, but at a slower rate, in subsequent years. Prevalence of osteoporosis in men receiving ADT for PCa is high. Over 50% of patients will suffer from osteoporosis if treated with ADT for 3 years and over 40% will have osteopenia [7]. All have increased risk of incident osteoporotic fractures [8]. Men with PCa receiving ADT thus represent a particularly vulnerable population at significant risk of falls and fractures.

For Australians over the age of 50, falls and fractures result in significant morbidity or even mortality, and are a considerable burden to the healthcare system and society [9]. Falls can have serious consequences such as major fracture (defined as major osteoporotic fracture [MOF] of hip, spine, lower and upper arm) [10] or head injury. Minor injuries such as bruising, lacerations, sprains and strains can still cause considerable pain, reduced function and fear of falling, and generate significant healthcare costs [11]. Exercise has an important role in managing many of the adverse effects of ADT for PCa [12], particularly in relation to key fall risk factors such as ADT-induced musculoskeletal changes [13], the potential to prevent fall-related fractures and injuries [14], as well as reduce fear of falling, a strong predictor of falls [15]. Recent exercise for cancer guidelines reported strong evidence to support improvements in physical function and moderate evidence to support improvements in bone health [16]. However, without any economic evaluations of exercise in this population, there is no economic evidence to support the implementation of such guidelines.

The purpose of this paper is to determine the cost-effectiveness of exercise in preventing falls and fractures in this high-risk population. A modelled cost-utility analysis was conducted to address the absence of available RCT evidence for men receiving ADT for PCa. Economic modelling is a timely and cost-effective method for providing decision makers with the information required to determine allocation of scarce resources. This study conforms to Consolidated Health Economic Evaluation Reporting Standards (CHEERS) [17] and economic modelling guidelines [18].

## Methods

### Population, perspective, time horizon and cycle length

The target population was individuals 65 years or older living in the community in Australia with a diagnosis of non-metastatic PCa (Stages I and II) receiving curative radiation therapy (RT) and adjuvant ADT, a population representative of the men expected to receive the exercise intervention. Based on this population, the mean age at commencement of the model was 68 [19].

The rationale behind the model is that exercise, comprising twice weekly group sessions of resistance, balance and functional training, supervised by an accredited exercise physiologist (AEP) or similarly qualified health professional, will reduce the risk of falls as well as the number of fractures and injuries sustained. These outcomes will translate into reduced health service use and hospitalisation, and improved quality of life. Given that Australia has a publicly funded healthcare system, a health system perspective was adopted to measure the cost per quality-adjusted life year (QALY) gain for the exercise intervention compared to usual care (advice to exercise only; no intervention).

The model consisted of two arms. The intervention arm was 12-month AEP supervised exercise training conducted for 1-h twice weekly in small groups of up to 10 participants. Training comprised a combination of moderate- to high-intensity resistance exercise using machines and aerobic exercise such as walking, cycling or jogging. Such interventions are effective in addressing the adverse effects of ADT for PCa [3, 4]. The comparator arm or usual care is exercise considered not to reduce falls [14] such as very gentle exercise, ‘sham’ exercise or a recommendation only to perform 150 min of moderate physical exercise per week [16, 20]. A 3-year time horizon for the economic model was deemed appropriate to capture the effect of 1 year of exercise training and an additional 2-year sustained effect of exercise in preventing falls [19, 21]. The cycle length was 3 months, the period of time generally required to recover from a fall injury or regain close to pre-fracture health-related utility [22].

### Model structure

**Fig. 1:**
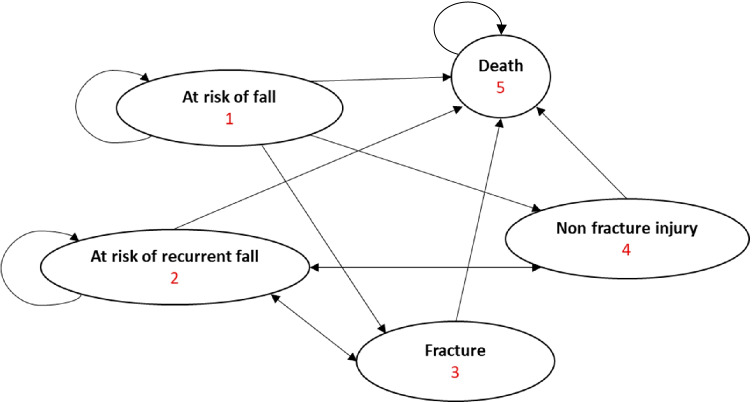
State transition diagram

The Markov model was designed to capture the natural transition between various health states. Individuals move between five Markov states in the model: (1) at risk of falling; (2) at recurrent risk of falling; (3) fracture; (4) non-fracture injury; and (5) death (Fig. 1). The fracture health state comprised two substates: minor and major fractures. Likewise, the non-fracture injury health state comprised both minor and major injuries.

All patients begin in the ‘at risk of falling’ state and remain there until they fall when they progress to fracture, non-fracture injury or death. Survivors then move to ‘at risk of recurrent fall’ state until they fall again, when they progress to fracture, non-fracture injury or death. Survivors then return to ‘at risk of recurrent fall’ each time after they fall.

### Model input parameters

Model input parameters comprise transition probabilities, costsand utilities and were derived from numerous sources (Table 1).

**Table 1:** Model parameters

Transition probabilities (12 months)	Distribution	Mean value	(95% CI)	Source
Fall in first year—control	Beta	0.36	(0.29, 0.43)	[5]
Recurrent fall in same year	Beta	0.65	(0.53, 0.77)	[5]
RR of fall in one year—exercise group	logNormal	0.76	(0.70, 0.81)	[14]
One or more fall-related fractures—control	Beta	0.12	(0.09, 0.15)	[14]
RR of one or more fall-related fractures—exercise	logNormal	0.44	(0.25, 0.76)	[14]
Major fracture (MOF)	Beta	0.62	(0.58, 0.66)	[8]
Minor fracture	Beta	0.38	(0.34, 0.42)	[8]
Non-fracture injury	Beta	0.88	(0.87, 0.89)	[14]
RR of non-fracture injury—exercise	logNormal	0.70	(0.54, 0.92)	[23]
Major non-fracture injury	Beta	0.06	(0.055, 0.065)	[8]
Minor non-fracture injury	Beta	0.94	(0.93, 0.95)	[8]
Death from fall	Beta	0.023 60–64yrs 0.043 65–69yrs 0.065 70–74yrs	(0.015, 0.031) (0.033, 0.053) (0.062, 0.068)	[24]
Age-related mortality		Table 60–75 yrs		[25]
Cost (12 months)				
Treatment major fracture	Gamma	$20,724	(20,082, 21,366)	[26]
Treatment for minor fracture	Gamma	$8,797	(8,524, 9,070)	[26]
Treatment major injury	Gamma	$10,040	(9,729, 10,351)	[27–29]
Treatment for minor injury (ED, non-admitted care, post discharge care)	Gamma	$1,115	(1,080, 1,150)	[28–30]
AEP supervised exercise intervention	Gamma	$767	(743, 791)	[30]
Utility				
Baseline pre-fracture/injury	Beta	0.79	(0.78, 0.80)	[31]
Major fracture (MOF)	Beta	0.475	(0.47, 0.49)	[22]
Minor fracture (‘non-hip, non-wrist, non-vertebral’)	Beta	0.565	(0.55, 0.59)	[22]
Major fall injury (not fracture)	Beta	0.47	(0.46, 0.48)	[32]
Minor fall injury/no injury (not fracture)	Beta	0.765	(0.76, 0.80)	[33]
Recurrent fall (FOF)	Beta	0.72	(0.70, 0.74)	[33]
Recurrent fall exercise (FOF)	Beta	0.74	(0.72, 0.76)	[34]

Abbreviations: RR*.,* relative risk; MOF*,* major osteoporotic fracture*—*hip, vertebrae, upper or lower arm; ED*,* emergency department; AEP*,* accredited exercise physiologist; FOF*,* fear of falling

#### Transition probabilities

Transition probabilities represent the probability of moving between the five states in the model and were based on published evidence of the highest level available. It was assumed that minor injuries or fractures do not cause death; major injuries or fractures may. Evidence for number of men who experienced a fall (health state 1) and men who experienced a recurrent fall in the same year (health state 2) was based on a study of falls and frailty in PCa survivors with data on current and past users of ADT [5]. Evidence from a recent systematic review of exercise for preventing falls in older people in the community was used to represent number of people experiencing fall-related fractures (health state 3) [14]. While this meta-analysis [14] refers to the general population of community-dwelling people 60 years and over, it provides high-level evidence where there was an absence of such evidence for PCa patients receiving ADT. Probabilities of non-fracture injury (health state 4), type of non-fracture injury (major and minor) and type of fracture (major and minor) were derived from evidence for patients with PCa receiving ADT in a large population-based cohort study [8]. Evidence for death (health state 5) in the population age groups of interest were based on Scuffham, Chaplin and Legood [24] for fall-related death and on Australian Bureau of Statistics Life Tables for age-related mortality [25]. Evidence for exercise in reducing the risk of falls, fall-related fractures and non-fracture injuries, was drawn from two meta-analyses [14, 23] (Table 1).

#### Costs

Costs were calculated for falls and consequent injury treatment. Assumptions made when calculating costs of treatment were as follows: a major injury or fracture refers to events requiring ED presentation and hospitalisation, followed by clinical and supportive care; minor fracture refers to a fracture requiring ED presentation and outpatient treatment in a hospital; minor injury refers to bruises, strains, cuts and sprains.

Cost of treatment for fractures, both minor and major, were based on Watts et al. [26] and converted to 2019 AUD. Costs for major injury (moderate TBI as proxy) were based on the approach used by Pavlov et al. [27] with Australian costs calculated from the Independent Hospital Pricing Authority (IHPA) National Efficient Price (NEP) 2019–2020 for hospital care [28] and costs of primary and community healthcare based on Hall and Hendrie [29] converted to 2019 AUD. Cost for treatment of minor injury was calculated over 3 months using the IHPA for hospital costs [28] and Hall and Hendrie [29] for primary and community healthcare costs. Given the vast difference in minor injuries and variation in the treatment required, it was assumed that at time of fall, 50% of fallers attend ED and are discharged after treatment; 25% see a GP and 25% do not seek medical treatment [35] (Table 1).

The cost of the exercise intervention was based on AEP led supervised training comprising two 1-h sessions per week over 1 year for men with localised or locally advanced PCa, estimated from a healthcare payer perspective. Implementation costs included labour for participant registration (Clerks private sector award), a pre-intervention consultation with an AEP (MBS no. 81110), conduct of exercise sessions of up to 10 people by an AEP (MBS no. 10953) and a GP visit (MBS no. 23) to determine eligibility for participation in exercise training. Services provided by the AEP and GP were valued using the Medical Benefits Schedule (MBS), a listing of services subsidised by the Australian government and part of the wider Medicare Benefits Scheme [30]. Exercise intervention cost was calculated with the assumption that cancer patients have access to 50 group sessions per year funded by the Australian government via MBS. Resource use costs included those costs specific to the intervention such as communication (telephone calls) with participants, material and printing costs (Table 2). Resources were valued using local or national costs where appropriate. All costs were reported in 2019 Australian dollars [36]. All other resource use categories were valued using market rates.

**Table 2:** Cost of exercise intervention (AU$2019)

Intervention cost component	Cost description	Unit of measure	Cost per participant
GP consent	MBS Item 23: Level B GP consultation lasting less than 20 min (2019)	1 consultation ($38.20)	$38
Registration of intervention participants & administration	Clerks private sector award 2010 level 3 $911/week ($23.97/h) + 20% on costs (2019)	30-min clerk time + phone calls	$15
AEP pre-program consultation	MBS Item no. 81110	1 consultation	$81
Subtotal			$134
50-week exercise intervention	1-h exercise session AEP MBS Item no. 10953	Up to 10 participants per session	$633
Total per participant (healthcare perspective)			$767

Abbreviations: *GP*, general practitioner; *MBS*, Medicare benefits schedule; *RCT*, randomised controlled trial; *AEP*, accredited exercise physiologist

#### Health state utilities

Health state utilities represent a preference value placed on a health state ranging from 1 for perfect health to 0 for death. Utility decrements reflect how an event such as a fall or fracture can impact negatively on a person’s health state. The resulting utility can then be used to calculate quality-adjusted life years (QALYs), where the utility represents the quality adjustment which is calculated over “life years” or the amount of time spent in that health state.

A baseline utility score representing the “well” state for men with PCa (pre-fall) was based on a population of men who had been receiving radiation therapy with adjuvant ADT for 2 months [31]. The health states in this study were measured using the Patient Oriented Prostate Utility Scale (PORPUS-U), a PCa-specific indirect utility instrument which was used to elicit standard gamble utilities (PORPUS-U_SG_) [31]_._

Fracture utilities were based on evidence from the Australian arm of the International Cost and Utility Related to Osteoporotic Fractures Study (AusICUROS) [22]. Health-related quality of life was estimated using the EuroQoL EQ-5D-3 L, a time trade-off (TTO) questionnaire. The values attached to each of the EQ-5D health states were based on TTO utility weights from general Australian population samples [22]. The utility value applied in the model for fracture was the mean of the utility score at time of fracture and the utility score at 3 months or one cycle in the Markov model. Utility for major fracture was based on major osteoporotic fracture (MOF) as defined in the Fracture Risk Assessment Tool (FRAX) (hip, vertebral, wrist or humerus fracture) [10]. Hip (40%) and vertebral fractures (30%) were the most common major fractures experienced by men with PCa receiving ADT [37] and constituted a fracture group in the AusICUROS study [22]. Utility for minor fractures was based on non-MOF fractures.

Utility for major non-fracture injury was based on a utility decrement for moderate traumatic brain injury (TBI), the second most common fall-related injury after hip fracture [32]. Utilities for minor non-fracture injury, recurrent falls and FOF were based on evidence from a study of falls and EQ-5D related quality of life of community-dwelling seniors with chronic diseases [33]. Exercise and the reduction in FOF were based on a systematic review of exercise to reduce FOF in older people living in the community [34] (Table 2).

### Cost-utility analysis

Costs and outcomes are represented in the model as the mean value per state per cycle. All 1-year input parameters will be converted to three monthly values for the four cycles of the Markov model with the exception of cost of treatment which was attributed in the first 3-month cycle after the fall event only, when the majority of costs are incurred. Costs and QALYs will be aggregated for the time horizon and compared between the intervention and control to calculate incremental net monetary benefit (iNMB) or the difference in quality-adjusted life years (QALYs) times the willingness-to-pay threshold (AU$50,000), minus the difference in costs. We set willingness-to-pay at $50,000 per QALY, a commonly used threshold for cost-effectiveness in Australia [38]. All costs and outcomes are discounted at a rate of 5% per year, a commonly applied rate in Australia [39]. Uncertainty in the model was explored via deterministic univariate and probabilistic sensitivity analysis. The analyses were conducted in TreeAge Pro Healthcare 2019 R1.1 and half-cycle corrections were used to adjust for overestimation of rewards in a traditional Markov model.

### Univariate sensitivity analysis

Assumptions were tested over a range of values using univariate deterministic sensitivity analyses to assess the robustness of the uncertainty in the parameter estimates including variation in intervention and health service costs, probability of occurrence of events and utility values (Table SI 1).

### Probabilistic sensitivity analysis

Probabilistic sensitivity analysis (PSA) involves random resampling of the model parameters followed by a recalculation of the NMB. The uncertainty around input parameters was modelled by fitting appropriate distributions to estimates obtained from the literature (Table 1). These were then used in a Monte Carlo simulation with 10,000 iterations to model joint parameter uncertainty. The results of the PSA are presented as a cost-effectiveness acceptability curve (CEAC) which plots the likelihood an intervention is cost-effective against a range of willingness-to-pay thresholds.

## Results

### Base-case analysis

At a willingness-to-pay of AU$50,000 per QALY gained, the exercise intervention dominated, as it was less costly and more effective than usual care. The exercise intervention was cost saving at $1183 less than usual care and the incremental effect was 0.04 QALYs gained. The iNMB of the exercise intervention was $3,010 per patient, suggesting that the intervention is cost-effective (Table 3).

**Table 3:** Results modelled CUA of supervised exercise intervention (12 months)

Variable	Control group	Intervention group	Difference	NMB		iNMB
Mean cost	$4,135	$2,952	$1,183	Control	$ 99,101	$3,010
Mean QALYs	2.06	2.10	0.04	Intervention	$102,112	

Abbreviations: *NMB*, net monetary benefit; *iNMB*, incremental net monetary benefit; *QALYs*, quality-adjusted life years

### Univariate sensitivity analyses

**Fig. 2:**
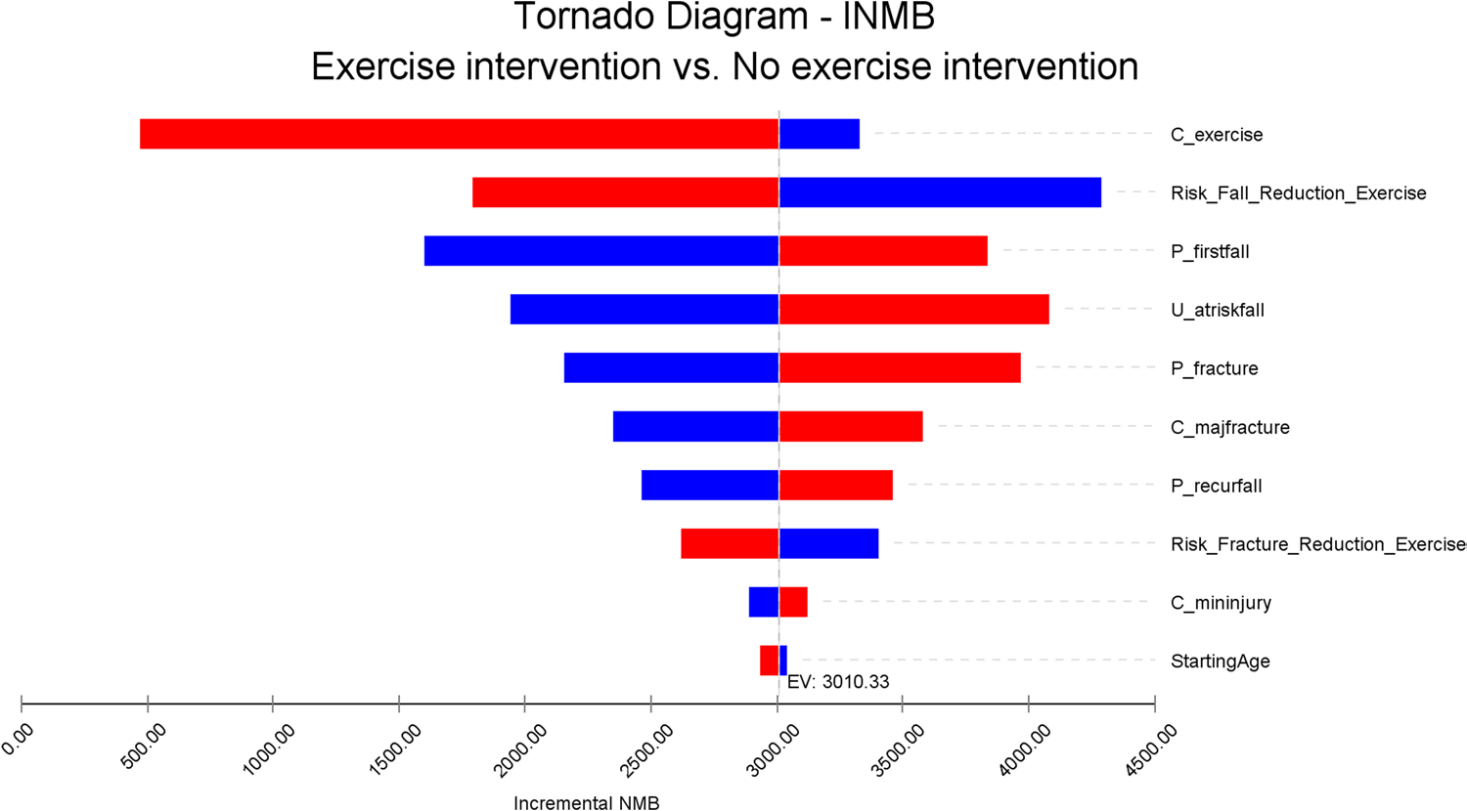
Univariate sensitivity analyses. Legend  Lower value of parameter  Higher value of parameter

The results of the univariate sensitivity analyses are shown in Fig. 2. The most sensitive parameters with the greatest influence on the iNMB were cost of exercise, exercise-induced fall risk reduction and probability of first fall. Even when the cost of exercise increases to amounts such as those in SA2a (12-month AEP supervised exercise for 6 people + per patient out of pocket (OOP) costs for travel and gym fees of $1150) ($2338), SA4 (a model-like group exercise for people with diabetes; MBS no. 81110) ($2154) and SA4a (SA4 + OOP costs as for SA2A) ($3304), the exercise intervention is cost-effective at a willingness-to-pay threshold of $50,000 per QALY gained (e.g. SA4a iNMB $474) (Table SI 1).

### Probabilistic sensitivity analysis

**Fig. 3:**
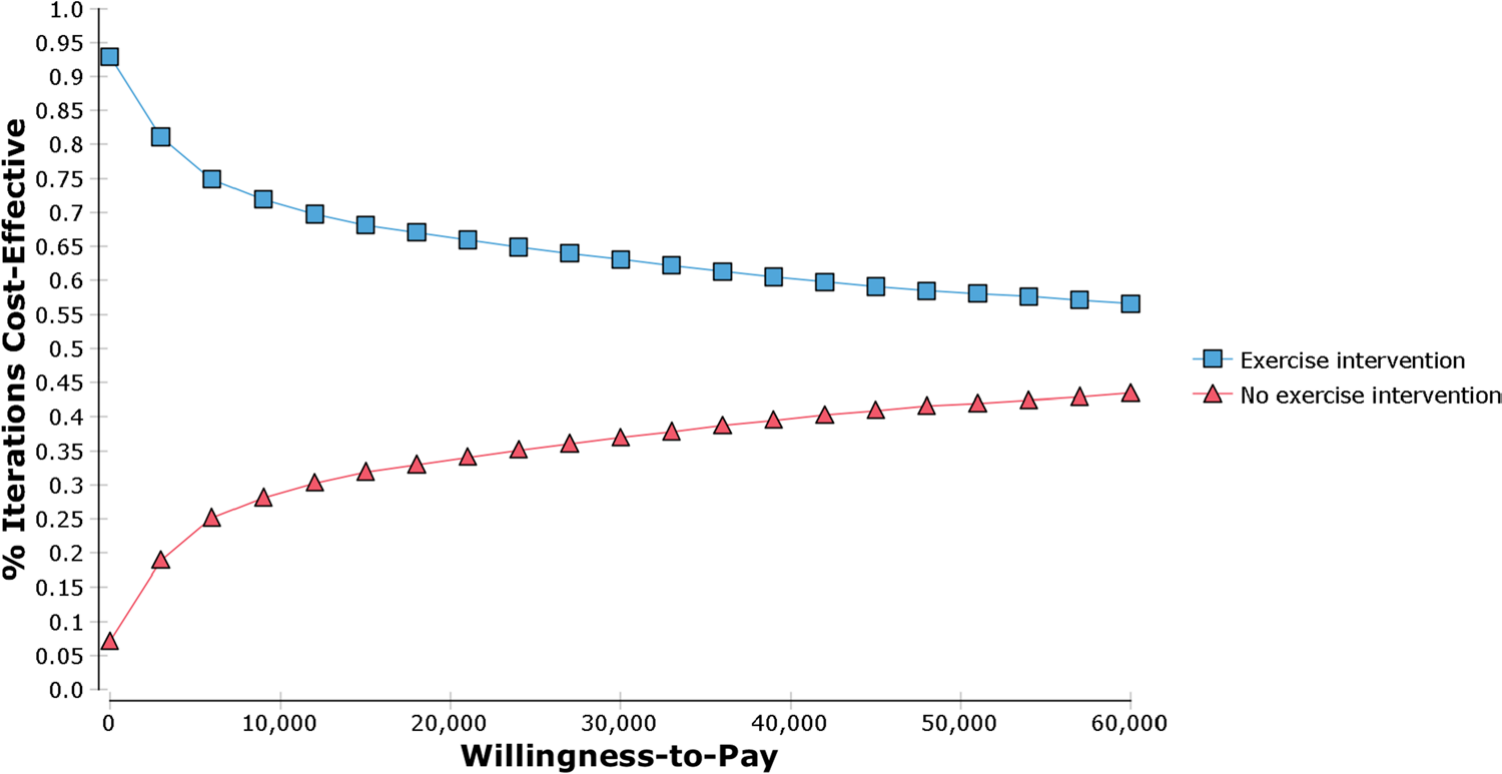
Cost-effectiveness acceptability curve

Probabilistic sensitivity analysis with 10,000 iterations of the parameter distributions resulted in a NMB of $102,085 (95%CI $101,808–$102,362). The probability that the intervention was cost-effective at a willingness-to-pay threshold of $50,000 per QALY gained was 58%. The cost-effectiveness acceptability curve (Fig. 3) shows that exercise compared to usual care will be cost saving over a range of willingness-to-pay values per QALY gained.

## Discussion

This is the first economic evaluation of exercise in preventing falls and fractures for men with PCa. The main finding indicates that exercise is cost saving at a willingness-to-pay threshold of $50,000 per QALY gained. The model suggests that even if exercise interventions are provided by the health system twice weekly for a year and patient OOP costs (gym membership and travel costs) are included, the intervention would be cost-effective. This is important information for policy makers when deciding which public health programs to support. Univariate sensitivity analyses showed the results were sensitive to the effectiveness of exercise in reducing risk of falls, the cost of the exercise intervention and probability of first fall. Probabilistic sensitivity analysis showed a 58% probability that the exercise intervention would be cost-effective at a willingness-to-pay of $50,000 per QALY gained.

A number of cost-effectiveness analyses of falls prevention exercise interventions for community dwelling older adults have been conducted, including both trial based [40, 41] and modelled or combined trial and model evaluations [42–45]. However, none included men with PCa and they varied considerably in terms of population age (stratified and not), fall risk, the interventions included (group or home-based exercise, nurse or AEP led, multi-factorial or multiple intervention), the comparators, outcomes measured and model structures. The two trial-based CEAs did not incorporate a multi attribute utility instrument (MAUI), so results were expressed as ICER per fall averted rather than QALYs gained, making comparison to our model impossible. The trial which used nurses to conduct group resistance and balance exercise training people aged 80 years and older was more cost-effective, with an ICER of $AU1219 (2019) per fall averted [41], than the multidisciplinary falls prevention program for people aged 70 years and older (including physiotherapy, occupational therapy, nurse, medical review and referral to other specialists) at AU$7679 (2019) per fall averted [40].

The results of this study are consistent with two of the modelled studies which were cost-effective in some form. One Markov model resulted in an ICER of AU$28,931 per QALY gained at a willingness-to-pay of AU$50,000, suggesting a public health intervention should be implemented. This result was based on a cost of $700 (2011 AUD) and a risk ratio for falls prevention of 0.75 for the general population aged 65 and over. The costs avoided of residential care admission, one arm of the model, would have contributed to the cost-effectiveness of this intervention [43]. The second model incorporated a care pathway (GP screening for falls risk) with two interventions, a home-based exercise program (Otago) and a group exercise program (FaME) [44]. The comparator was no care pathway. Results were stratified for age. FaME was dominant for ages 65–89, whereas Otago was dominant in the 75–89 age group, but cost-effective for the 70–89 age group. In the other two models, group-based exercise was only cost-effective in the women only program in one study [45] and neither home-based nor group-based exercise was cost-effective in the other [42]. Differences tend to derive from model structure. Only the FaME program achieved similar results to our study, but in a slightly older age group (70–89 vs 65–75). This is possibly because men with PCa receiving ADT are at higher risk than the general population of a similar age. The fact that our model included costs for all injuries treated, regardless of severity, may also have contributed to exercise being dominant in most scenarios analysed.

The results of this modelled study indicate that a public health program of AEP supervised exercise for fall prevention should be implemented for men with PCa who are receiving or have received ADT. A systematic review of exercise to prevent falls and fractures in older community-dwelling people found that functional and balance exercise supervised by health professionals (e.g. AEP and physiotherapists) is more effective than unsupervised exercise in reducing rate of falls [14]. Having access to this expertise is particularly important for men with PCa who may have been impacted by the adverse effects of ADT and at a higher risk of falls and fractures than the general population.

### Strengths and limitations

The strengths of this modelled evaluation are the use of QALYs as an outcome measure enabling policy makers to make comparisons across different health programs. The model structure reflects a realistic fall scenario by incorporating transition probabilities for falls, recurrent falls, utility decrements for fear of falling and a range of fall consequences such as fall-related fractures and non-fracture injuries, both major and minor. The time horizon is relatively short and based on only 1 year of supervised exercise. However, sensitivity analyses doubling the time horizon to a 6-year time frame almost doubled the NMB and the exercise intervention maintained its dominance. Incorporation of longer follow-up to collect data on the impact of ADT-induced metabolic changes such as diabetes, cardiac and vascular disorders, for example, and their associated treatment costs is likely to contribute to more cost-effective outcomes. Men similar to the population in this study can maintain the benefits of 6 months supervised exercise with home-based exercise [19]. Many men find the health and wellbeing benefits, camaraderie and masculinity enhancing aspects of group exercise rewarding and continue to exercise beyond intervention timelines [46]. For these men, the time horizon for exercise and the associated benefits would be extended, potentially enhancing cost-effectiveness. This would also suggest the results of our model are conservative.

One limitation is the costing of an Australian intervention, which may not translate to exercise programs in other countries. However, when sensitivity analyses increased exercise program costs to over $3000 ($3304), the exercise intervention was still cost-effective at a willingness-to-pay threshold of $50,000 (iNMB $474). Another limitation is that not all model inputs were drawn from the PCa population. Where there is an absence of individual level patient data, models must utilise numerous sources to derive evidence. In the absence of evidence for men with PCa, evidence from comparable populations and from the highest level sources available [14, 23, 34] were used.

## Conclusion

This is the first cost-utility analysis of exercise in preventing falls and fractures for men with PCa treated with ADT. Supervised exercise is likely to improve quality of life and be cost saving in this vulnerable population. These findings strongly suggest that a public health program of AEP led exercise for falls prevention should be implemented for men with PCa who are receiving or have received ADT. This model structure could also have application in the modelling of falls in other populations, such as other cancer or disease groups, different age groups or the general population, if updated with appropriate model input parameters.

## Supplementary Information

Below is the link to the electronic supplementary material.

Supplementary file1 (DOCX 24 KB)

## Data Availability

Not applicable.

## References

[1] Australian Institute of Health and Welfare (2019) Cancer in Australia 2019. Canberra

[2] Australian Institute of Health and Welfare (2014) Cancer in Australia: an overview 2014. Cancer series No 90. Cat. no. CAN 88. AIHW, Canberra

[3] Galvão D, Taaffe D, Spry N, Joseph D, Turner D, Newton R (2009). Reduced muscle strength and functional performance in men with prostate cancer undergoing androgen suppression: a comprehensive cross-sectional investigation. Prostate Cancer Prostatic Dis.

[4] Newton RU, Jeffery E, Galvão DA, Peddle-McIntyre CJ, Spry N, Joseph D (2018). Body composition, fatigue and exercise in patients with prostate cancer undergoing androgen-deprivation therapy. BJU Int.

[5] Winters-Stone K, Moe E, Graff J, Dieckmann N, Stoyles S, Borsch C (2017). Falls and frailty in prostate cancer survivors: current, past and never users of androgen therapy. J Am Geriatr Soc.

[6] Grossmann M, Hamilton EJ, Gilfillan C, Bolton D, Joon DL, Zajac JD (2011). Bone and metabolic health in patients with non-metastatic prostate cancer who are receiving androgen deprivation therapy. Med J Aust.

[7] Lassemillante A, Doi S, Hooper J, Prins J, Wright O (2014). Prevalence of osteoporosis in prostate cancer survivors: a meta-analysis. Endocrine.

[8] Wallander M, Axelsson K, Lundh D, Lorentzon M (2019). Patients wth prostate cancer and androgen deprivation therapy have increased risk of fractures-a study from the fractures and fall injuries in the elderly cohort (FRAILCO). Osteoporos Int.

[9] Kreisfeld R, Pointer S, Bradley C (2017). Trends in hospitalisations due to falls by older people, Australia: 2002–03 to 2012–13.

[10] Kanis J, Johnell O, Oden A, Johansson H, McKloskey E (2008). FRAX and the assessment of fracture probability in men and women from the UKFracture Risk Assessment Tool. Osteoporos Int.

[11] Burns E, Stevens J, Lee R (2016). The direct costs of fatal and non-fatal falls among older adults - United States. J Saf Res.

[12] Edmunds K, Tuffaha H, Scuffham P, Galvão D, Newton R (2020). The role of exercise in the management of adverse effects of androgen deprivation therapy for prostate cancer: a rapid review. J Support Care Cancer.

[13] Newton RU, Galvão DA, Spry N, Joseph D, Chambers SK, Gardiner RA (2018). Exercise mode specificity for preserving spine and hip bone mineral density in prostate cancer patients. Med Sci Sports Exerc.

[14] Sherrington C, Fairhall N, Wallbank G, Tiedemann A, Michaleff Z, Howard K et al (2019) Exercise for preventing falls in older people living in the community (review). Cochrane Database Sys Rev. Art No.: CD012424(1) 10.1002/14651858.CD012424.pub2 PMC6360922 30703272

[15] Kendrick D, Kumar A, Carpenter H, Zijlstra G, Skelton D, Cook J et al (2014) Exercise for reducing fear of falling in older people living in the community. Cochrane Database Syst Rev (11) 10.1002/14651858.CD009848.pub2 PMC7388865 25432016

[16] Campbell K, Winters-Stone K, Wiskemann J, May A, Schwartz A, Courneya K (2019). Exercise guidelines for cancer survivors: consensus statement from International Multidisciplinary Roundtable. Med Sci Sports Exerc.

[17] Husereau D, Drummond M, Petrou S, Carswell C, Moher D, Greenberg D (2013). Consolidated health economic evaluation reporting standards (CHEERS)-explanation and elaboration: a report of the ISPOR health economic evaluation publication guidelines good reporting practices task force. Value Health.

[18] Caro J, Briggs A, Siebert U, Kuntz K, Force oBotI-SMGRPT (2012). Modeling good research practices-overview: a report of the ISPOR-SMDM Modeling Good research Practices Task Force-1. Value Health.

[19] Galvão DA, Spry N, Denham J, Taaffe DR, Cormie P, Joseph D (2014). A multicentre year-long randomised controlled trial of exercise training targeting physical functioning in men with prostate cancer previously treated with androgen suppression and radiation from TROG 03.04 RADAR. Eur Urol.

[20] Hayes S, Newton R, Spence R, Galvão D (2019). The Exercise and Sports Science Australia position statement: exercise medicine in cancer management. J Sci Med Sport.

[21] Finnegan S, Seers K, Bruce J (2019). Long-term follow-up of exercise interventions aimed at preventing falls in older peopleliving in the community: a systematic review and meta-analysis. Physiotherapy.

[22] Abimanyi-Ochom J, Watts J, Borgström F, Nicholson G, Shore-Lorenti C, Stuart A (2015). Changes in quality of life associated with fragility fractures: Australian arm of the International Cost and Utility Related to Osteoporotic Fractures Study (ICUROS). Osteoporos Int.

[23] El-Khoury F, Cassou B, Charles M-A, Dargent-Molina P (2013) The effect of fall prevention exercise programmes on fall induced injuries in community dwelling older adults: systematic review and meta-analysis of randomised controlled trials. BMJ (Online) 347 10.1136/bmj.f6234 PMC3812467 24169944

[24] Scuffham P, Chaplin S, Legood R (2003). Incidence and costs of unintentional falls in older people in the United Kingdom. J Epidemiol Community Health.

[25] Australian Bureau of Statistics (2018) Life Tables. States, Territories and Australia, 2016–2018, cat. no. 3302.0.55.001. In: Statistics ABo, editor. Canberra: Australian Bureau of Statistics

[26] Watts J, Abimanyi-Ochom J, Sanders K (2013). Osteoporosis costing all Australians a new burden of disease analysis – 2012 to 2022.

[27] Pavlov V, Thompson-Leduc P, Zimmer L, Wren J, Shea J, Beyhaghi H (2019). Mild traumatic brain injury in the United States: demographics, brain imaging procedures, health-care utilization and costs. Brain Inj.

[28] Independent Hospital Pricing Authority IHPA (2019) National Efficient Price Determination 2019–20 Sydney: IHPA. Available from: https://www.ihpa.gov.au/publications/national-efficient-price-determination-2019-20. Accessed 30 Aug 2020

[29] Hall S, Hendrie D (2003). A prospective study of the costs of falls in older adults living in the community. Aust N Z J Public Health.

[30] Department of Health (2017) MBS Online: Medicare Benefits Schedule Canberra: Australian Government. Available from: http://www.mbsonline.gov.au/internet/mbsonline/publishing.nsf/Content/Home. Accessed 28 Aug 2020

[31] Krahn MD, Bremner KE, Tomlinson G, Naglie G (2009). Utility and health-related quality of life in prostate cancer patients 12 months after radical prostatectomy or radiation therapy. Prostate Cancer Prostatic Dis.

[32] Dijkers M (2004). Quality of life after traumatic brain injury: a review of research approaches and findings. Arch Phys Med Rehabil.

[33] Thiem U, Klaaßen-Mielke R, Trampisch U, Moschny A, Pientka L, Hinrichs T (2014) Falls and EQ-5D rated quality of life in community-dwelling seniors with concurrent chronic diseases: a cross-sectional study. Health Qual Life Outcomes 12(2) 10.1186/1477-7525-12-2 PMC3895701 24400663

[34] Kumar A, Delbaere K, Zijlstra G, Carpenter H, Iliffe S, Masud T (2016). Exercise for reducing fear of falling in older people living in the community: Cochrane systematic review and meta-analysis. Age Ageing.

[35] Stevens J, Ballesteros M, Macl K, Rudd R, DeCaro E, Adler G (2012). Gender differences in seeking care for falls in the aged medicare population. Am J Prev Med.

[36] Organization for Economic Co-operation and Development (OECD) (2018) GDP implicit price deflator St Louis: FRED, Federal Reserve Bank of St Louis. Available from: https://fred.stlouisfed.org/series/. Accessed 2 Nov 2020

[37] Shahinian V, Kuo Y, Freeman J, Goodwin J (2005). Risk of fracture after androgen deprivation for prostate cancer. N Engl J Med.

[38] Henry D, Hill S, Harris A (2005). Drug prices and value for money. JAMA.

[39] Pharmaceutical Benefits Advisory Committe (PBAC) (2016) Guidelines for preparing submissions to the Pharmaceutical Benefits Advisory Committee (PBAC) Version 5 Canberra: Department of Health. Available from: https://pbac.pbs.gov.au/. Accessed 19 Oct 2020

[40] Irvine L, Conroy S, Sach T, Gladman J, Harwood R, Kendrick D (2010). Cost-effectiveness of a day hospital falls prevention programme for screened community-dwelling older people at high risk of falls. Age Ageing.

[41] Robertson M, Gardner M, Devlin N, McGee R, Campbell J (2001). Effectiveness and economic evaluation of a nurse delivered home exercise programme to prevent falls. 2: controlled trial in multiple centres. Br Med J.

[42] Church J, Goodall S, Norman R, Haas M (2011). The cost-effectivenes of falls prevention interventions for older community-dwelling Australians. Aust N Z J Public Health.

[43] Farag I, Howard K, Ferreira M, Sherrington C (2015). Economic modelling of a public health programme for fall prevention. Age Ageing.

[44] Franklin M, Hunter R (2020). A modelling-based economic evaluation of primary-care-based fall-risk screening followed by fall-prevention intervention: a cohort-based Markov model stratified by older age groups. Age Ageing.

[45] McLean K, Day L, Dalton A (2015) Economic evaluation of a group-based exercise program for falls prevention among the older community-dwelling population. BMC Geriatrics 15 10.1186/s12877-015-0028-x PMC4404560 25879871

[46] Cormie P, Oliffe JL, Wootten AC, Galvão DA, Newton RU, Chambers SK (2016). Improving psychosocial health in men with prostate cancer through an intervention that reinforces masculine values – exercise. Psychooncology.

