# Supplementary material for: Exercise in preventing falls for men with prostate cancer: a modelled cost-utility analysis
Source: Support Care Cancer. 2022 Feb 24;30(6):5037–46. doi: 10.1007/s00520-022-06900-2 (PMC9046330; doi:10.1007/s00520-022-06900-2)
Supplement: Supplementary file 1 — Supplementary file1 (DOCX 24 KB) [file 520_2022_6900_MOESM1_ESM.docx]

# Supplementary Information

## **Exercise in preventing falls for men with prostate cancer receiving androgen: a modelled cost-utility analysis**

# Journal of Supportive Care in Cancer

Kim Edmunds, Paul Scuffham, Robert U Newton, Daniel A Galvão, Haitham Tuffaha

Corresponding Author: Kim Edmunds, University of Queensland, k.edmunds@uq.edu.au

#

# Table SI1 Univariate sensitivity analysis

| **Variable** | **Strategy** | **Inc. Cost** | **Inc. QALY** | **iNMB** |
| --- | --- | --- | --- | --- |
| **Base C_exercise**  $767 Base - 12mos AEP (MBS no. 10953) -health service | Exercise intervention  (10 persons) | -$1,183 | 0.04 | $3,011 |
| **SA1a C_exercise**  $1917 Base - 12mos AEP (MBS no. 10953) -part societal | Exercise intervention  (10 persons) + OOP costs | -$33 | 0.04 | $1,861 |
| **SA2 C_exercise**  $1188 - 12mos AEP (MBS no. 10953 )  -health service | Exercise intervention  (6 persons) | -$762 | 0.04 | $2,590 |
| **SA2a C_exercise**  $2338 - 12mos AEP  (MBS no. 10953)-part societal | Exercise intervention  (6 persons) + OOP costs | $388 | 0.04 | $1,440 |
| **SA3 C_exercise**  $450 6mos AEP  (MBS no. 10953) + 6mos home-based  -health service | Exercise intervention  6mos + 6mos home-based  (10 persons) | -$1,500 | 0.04 | $3,328 |
| **SA3a C_exercise**  $709 - 6mos AEP (MBS no. 10953)  + 6mos home-based-part societal | Exercise intervention  6mos + 6mos + OOP costs  (10 persons) | -$1,241 | 0.04 | $3,069 |
| **SA4 C_exercise**  $2154 12mos AEP group diabetes  (MBS no. 81110)-health service | Exercise intervention diabetes  (10 persons) | $204 | 0.04 | $1,624 |
| **SA4a C_exercise**  $3304 12mos AEP group diabetes  (MBS no. 81110)-part societal | Exercise intervention  diabetes + OOP costs  (10 persons) | $1,354 | 0.04 | $474 |
| **SA5 C_majfracture**  Mean -50% +50% ($10K-$30K) | Exercise intervention  (10 persons) | -$527  -$1,750 | 0.04 | $2354  $3,578 |
| **SA6-C_mininjury**  Mean -50% +50% ($0.55K-$1.65K) | Exercise intervention  (10 persons) | -$1,064  -$1296 | 0.04 | $2,891  $3,123 |
| **SA7 P_Risk_Fall_Reduction**  Mean -0.2 +0.2 (RR 0.56-0.96) | Exercise intervention  (10 persons) | -$1,797  -$564 | 0.5  0.02 | $4,290  $1,795 |
| **SA8 P_Risk_Fracture_Reduction**  Mean -0.2 +0.2 (RR 0.24-0.64) | Exercise intervention  (10 persons) | -$1,505  -$861 | 0.04 | $3,403  $2,619 |
| **SA9 P_firstfall**  Mean -/+50% (0.0525-0.1575) | Exercise intervention  (10 persons) | -$645  -$2,085 | 0.02  0.05 | $1,846  $4,380 |
| **SA10 P_recurfall**  Mean -/+50% (0.1145-0.3435) | Exercise intervention  (10 persons) | -$1,765  -$928 | 0.04  0.03 | $3,767  $2,599 |
| **SA11-P_fracture**  Mean -/+50% (0.06-0.18) | Exercise intervention  (10 persons) | -$646  -2467 | 0.03  0.04 | $2,357  $4,574 |
| **SA12 U_atriskfall**  Mean -0.1 +0.1 (0.69-0.89) | Exercise intervention  (10 persons) | -$1505 | 0.02  0.06 | $2,332  $4472 |
| **SA13 StartingAge**  Mean -8 yrs +8 yrs (60-76) | Exercise intervention  (10 persons) | -$1531  -$1461 | 0.04  0.04 | $3436  $3318 |
| **SA14 Time horizon (total cycles)**  6 yrs (24) | Exercise intervention  (10 persons) | -$4216 | 0.09 | $8,625 |

Abbreviations: Inc. cost incremental cost; Inc. QALY incremental quality adjusted life year; INMB incremental net monetary benefit;

SA-C sensitivity analysis cost; SA-P sensitivity analysis probability; SA-U sensitivity analysis utility; yrs years
